# An Evaluation of the Effect of Opioid-Free Anesthesia on Early Quality of Recovery in Patients Undergoing Gynecological Cancer Surgeries: A Randomized Controlled Trial

**DOI:** 10.7759/cureus.114038

**Published:** 2026-08-05

**Authors:** Malini Kanchan, Geetanjali Chilkoti, Swati Jain, Medha Mohta, Sanjna A Tiwari, Puneet Chugh, Michell Gulabani, Priyanka Sinha, Md Shakir

**Affiliations:** 1 Department of Anaesthesiology and Critical Care, University College of Medical Sciences and GTB Hospital, Delhi, IND

**Keywords:** cancer surgeries, dexmedetomidine, ketamine, opioid, quality of recovery

## Abstract

Background and objective

While quality of recovery (QoR) has been studied in breast cancer surgeries using opioid-free anesthesia (OFA), it has not been assessed as a primary outcome in gynecological cancer surgeries. This study aimed to evaluate QoR using the QoR-40 score in gynecological cancer surgeries, comparing OFA with opioid-based anesthesia (OBA). Secondary outcomes included pain scores, requirement for rescue analgesia, postoperative nausea and vomiting (PONV), hemodynamic parameters, time to first ambulation, DN-4 scores, and length of hospital stay.

Methods

After obtaining Institutional Ethics Committee (IEC) approval and registration with the Clinical Trials Registry-India (CTRI), the study enrolled American Society of Anesthesiologists Physical Status (ASA-PS) I-II patients aged 20-65 years undergoing exploratory laparotomy for gynecological cancer. Exclusion criteria included chronic pain, a history of PONV, allergy to local anesthetics, coagulopathy, opioid use, or cognitive dysfunction. Group OF received dexmedetomidine and ketamine infusions, whereas group OB received IV morphine. All patients underwent epidural catheter placement and general anesthesia according to the study protocol, with epidural analgesia continued for 48 hours. The aforementioned secondary outcomes were recorded at 12 hours and on postoperative days one, two, and three.

Results

Eighty patients were enrolled in the study, with 40 patients in each group and comparable demographics. QoR was significantly better with OFA than with OBA. PONV was significantly lower in the OFA group for up to eight hours postoperatively. No significant differences were found in Numerical Rating Scale (NRS)-Pain scores, rescue analgesic use, time to ambulation, oral fluid tolerance, or hospital stay.

Conclusions

OFA significantly improved postoperative QoR and analgesia, and reduced PONV in patients undergoing gynecological cancer surgery, while intraoperative and postoperative hemodynamics, ambulation, oral fluid tolerance, and hospital stay remained comparable between groups.

## Introduction

Opioids have traditionally been the mainstay for providing analgesia in the perioperative period. Concerns regarding their use include various opioid-related adverse drug events (ORADE), such as nausea, vomiting, somnolence, urinary retention, constipation, hypotension, respiratory depression/apnoea, bradycardia, and others. This has prompted the judicious use of opioids in the perioperative period [1-3].

Opioid-free anesthesia (OFA) is defined as the combination of various opioid-sparing strategies that culminate in the complete avoidance of perioperative opioid use to reduce ORADE without affecting patient comfort [1-3]. OFA was administered using a multimodal combination of non-opioid adjuvants comprising dexmedetomidine, ketamine, lidocaine, magnesium sulphate, paracetamol, nonsteroidal anti-inflammatory drugs (NSAIDs), and epidural analgesia, without the use of intraoperative opioids [3]. The concept of OFA has also been embraced in various cancer surgeries and is equally efficacious to conventional opioid-based anesthesia (OBA) using intraoperative IV opioids [4-6].

The measurement of quality of recovery (QoR) following anesthesia and surgery involves assessing the patient’s postoperative status in comparison with their preoperative status and includes the return of self-care, household and work activities, and mobility to preoperative levels. In measuring patient outcomes, there is a clear trend toward the use of patient-reported outcome measures (PROMs), i.e., the direct reporting of an individual’s health status by the individual [7]. Our literature search revealed that QoR assessment as a PROM using an OFA regimen had been performed in breast cancer surgeries: Hontoir et al. [8] and Zhang et al. [9] had used QoR-40 and QoR-15 scores, respectively. Considering that PROMs are increasingly recognized as important postoperative outcomes, and that both pain intensity and OFA protocols vary across different cancer surgeries, we found no study evaluating QoR scores as the primary outcome following OFA in gynecological cancer surgeries.

Thus, we undertook the present study to evaluate QoR using the QoR-40 score in patients receiving OFA compared with conventional OBA for gynecological cancer surgeries, with PROMs in the form of the QoR score serving as the primary measure for assessing the therapeutic efficacy of OFA. The secondary outcomes included NRS pain scores, rescue analgesic consumption, incidence of postoperative nausea and vomiting (PONV), hemodynamics, time to first independent ambulation, and total hospital stay.

## Materials and methods

This prospective randomized controlled study was conducted at a tertiary care center following approval from the Institutional Ethics Committee (vide approval no. IECHR-2022-55-74) and was subsequently registered with the Clinical Trials Registry-India (CTRI) (vide approval no. CTRI/2022/12/048126). The study was carried out in accordance with the principles of the Declaration of Helsinki 2013 and confirmed that patient data would be used for research and educational purposes. Written informed consent was obtained from all participants before their recruitment.

Consenting female patients with American Society of Anesthesiologists Physical Status (ASA-PS) I-II, as defined by the American Society of Anesthesiologists [10], aged 20-65 years, undergoing exploratory laparotomy for gynecological cancer surgeries, including radical hysterectomy, staging laparotomy, ovarian cancer debulking surgery, and endometrial cancer staging procedures, were included. Exclusion criteria included patients with a history of chronic pain of any etiology, PONV, previous allergy to local anesthetics, or coagulopathies; patients receiving opioids; and patients with a potential inability to comprehend questionnaires.

Quality of recovery was assessed using the validated 40-item Quality of Recovery questionnaire (QoR-40) developed by Myles et al. [11]. All patients completed the QoR-40 questionnaire at baseline and postoperatively at 12 hours, postoperative day one, and postoperative day three. The questionnaire consists of 12 questions measuring the comfort state; nine questions measuring the emotional state; seven questions measuring the psychological state; five questions measuring physical independence; and seven questions measuring the level of pain. Each question was assigned a score of 1-5, with a minimum possible score of 40 and a maximum possible score of 200. For patients requiring assistance, a blinded trained assessor read the questions verbatim and recorded responses without providing clarification or prompting to avoid interviewer bias.

Before its use, the original English QoR-40 questionnaire was translated and culturally adapted into Hindi using a standardized five-step process to ensure cultural relevance and linguistic accuracy [12]. The steps included forward translation by two independent translators (T1 and T2), synthesis into a single version (T12) with resolution of minor discrepancies, such as the term “nausea,” back translation into English by two translators, including one expert, to ensure conceptual equivalence, expert committee review to finalize the pretest version, and pretesting in 10 randomly selected postoperative patients. The Hindi version (QoR-40 H) was found to be clear, culturally appropriate, and easily comprehensible, with no modifications required before its final approval by the expert committee.

Patients were randomly allocated into one of the two groups, i.e., group OF (opioid-free) and group OB (opioid-based). Randomization was performed using a computer-generated random number table, and allocation concealment was achieved using opaque, serially numbered, sealed envelopes. In all patients, an epidural catheter was inserted preoperatively, followed by GA as per the standard institutional protocol. Standard ASA-recommended minimal mandatory monitoring of patients, including continuous ECG, heart rate (HR), pulse oximetry, end-tidal CO₂ (EtCO₂), core body temperature, and intermittent non-invasive blood pressure monitoring, was instituted and recorded.

Patients received dual antiemetic therapy with 4 mg of ondansetron and 8 mg of dexamethasone. Intraoperative hemodynamic parameters were recorded at baseline, five minutes after induction of anesthesia, and subsequently at 10-minute intervals until the end of surgery. The number of episodes of hypotension (systolic blood pressure (SBP) decrease of more than 20% from baseline) and bradycardia (heart rate less than 45 bpm) were recorded and managed with vasopressors and atropine, respectively. All patients received 1 g of intravenous paracetamol approximately 15 minutes before tracheal extubation. In both groups, epidural analgesia was administered intraoperatively using bupivacaine 0.125-0.25%, with intermittent bolus doses of 6-8 mL based on hemodynamic response and analgesic requirements.

Patients in group OF received dexmedetomidine 1 μg/kg over 10 minutes before induction of anesthesia. Anesthesia was induced with propofol 1-2 mg/kg IV and vecuronium 0.08-0.1 mg/kg IV. Anesthesia was maintained with O₂, N₂O, isoflurane, and vecuronium top-ups. Intraoperative analgesia was maintained with intermittent epidural top-up doses of bupivacaine (6-8 mL of 0.125-0.25% bupivacaine) and IV infusions of dexmedetomidine at a rate of 0.3-0.7 mcg/kg/hr and ketamine at a rate of 0.25-0.5 mg/kg/hr. Both infusions were stopped approximately 30 minutes before the completion of surgery. A 20% rise in mean arterial pressure (MAP) or HR prompted titration of the dexmedetomidine and ketamine infusions or administration of an epidural top-up dose. If these changes persisted, the use of opioids, i.e., fentanyl 1-2 mcg/kg, was considered. Patients in group OB received standard anesthesia as per institutional protocol. Anesthesia was induced with propofol 1-2 mg/kg IV, morphine 0.1 mg/kg IV, and vecuronium 0.08-0.1 mg/kg IV. An additional 3-6 mg bolus of morphine was administered if a 20% rise in HR or SBP was observed during the procedure.

In both groups, isoflurane concentration was titrated between 0.8-1.2 minimum alveolar concentration (MAC) to maintain adequate anesthetic depth. Nitrous oxide was administered in a 50:50 mixture with oxygen. Total intraoperative anesthetic exposure, including volatile anesthetic concentration, was guided by hemodynamic parameters as per the institutional protocol. Postoperatively, all patients in both groups received inj. paracetamol (PCM) 1 g and intermittent epidural top-ups of 6-8 mL of 0.125% bupivacaine every eight hours following confirmation of adequate catheter function and hemodynamic stability. If the visual analogue scale (VAS) was ≥4 despite round-the-clock PCM and epidural top-ups, rescue analgesia in the form of inj. diclofenac 50 mg IV was administered.

All patients completed the QoR-40 questionnaire postoperatively at the 12th hour, day one, and third day. The rescue analgesic consumption of diclofenac 50 mg IV over 24 hours was recorded. Postoperative nausea and vomiting, time to first independent ambulation (in minutes), and time to tolerate oral fluids (in minutes) were recorded. Pain assessment using the Numerical Rating Scale (NRS) was recorded at the second hour, fourth hour, ninth hour, 12th hour, and at the end of day one. An investigator trained in administering the QoR-40 questionnaire and blinded to group allocation assisted all patients in completing the QoR-40 questionnaire at the 12th hour, day one, and day three after the patient arrived in the post-anesthesia care unit. PONV was defined as any nausea, retching, or vomiting occurring during the first 24 hours after surgery. In the post-anesthesia care unit and the ward, patients were asked to report nausea or an episode of retching or vomiting in a yes/no format at four-hourly intervals. Rescue antiemetic therapy with metoclopramide 10 mg IV was administered when required.

The primary outcome was the QoR-40 score at the 12th hour, day one, and day three. Pain intensity was measured using the 11-point NRS (NRS; 0 = no pain and 10 = worst imaginable pain), a validated tool for acute pain assessment [13]. NRS pain scores were assessed at the second hour, fourth hour, ninth hour, 12th hour, and at the end of day one. Other secondary outcomes included PONV incidence during the first 24 hours, rescue analgesic consumption during the first 24 hours, time to first independent ambulation, time to tolerate oral fluids, and intraoperative hemodynamic parameters.

The sample size was calculated assuming a clinically significant difference of 5% in the mean QoR-40 score between the OFA and OBA groups. Based on a previous study, the mean (standard deviation (SD)) QoR-40 score at 24 hours after pelvic gynecological surgery was 173 (13.33) [14]. Assuming a two-sided α error of 0.05 and a study power of 80%, a minimum sample size of 34 patients per group (68 patients in total) was required. To account for potential dropouts, the final sample size was increased to 80 patients, with 40 patients assigned to each group.

The data were analyzed using SPSS Statistics version 31 (IBM Corp., Armonk, NY). The quantitative variables were reported with mean (SD) or median (interquartile range (IQR)), and qualitative variables with number and percentage. Normality was tested using a box-whisker plot and also the Shapiro-Wilk test. The unpaired Student t-test was applied for normally distributed variables and the Mann-Whitney U test for the non-normally distributed variables. The Chi-square and Fisher’s exact test were used for the qualitative variables. A linear mixed model with a suitable covariance structure was performed to compare the hemodynamic variables, and Bonferroni adjustment was applied to control type-I error. The primary outcome, QoR-40 score, measured at multiple postoperative time points (12 hours, day one, and day three), was analyzed using a linear mixed-effects model to account for within-subject correlations arising from repeated measurements. A p-value less than 0.05 was considered statistically significant.

## Results

**Figure 1:**
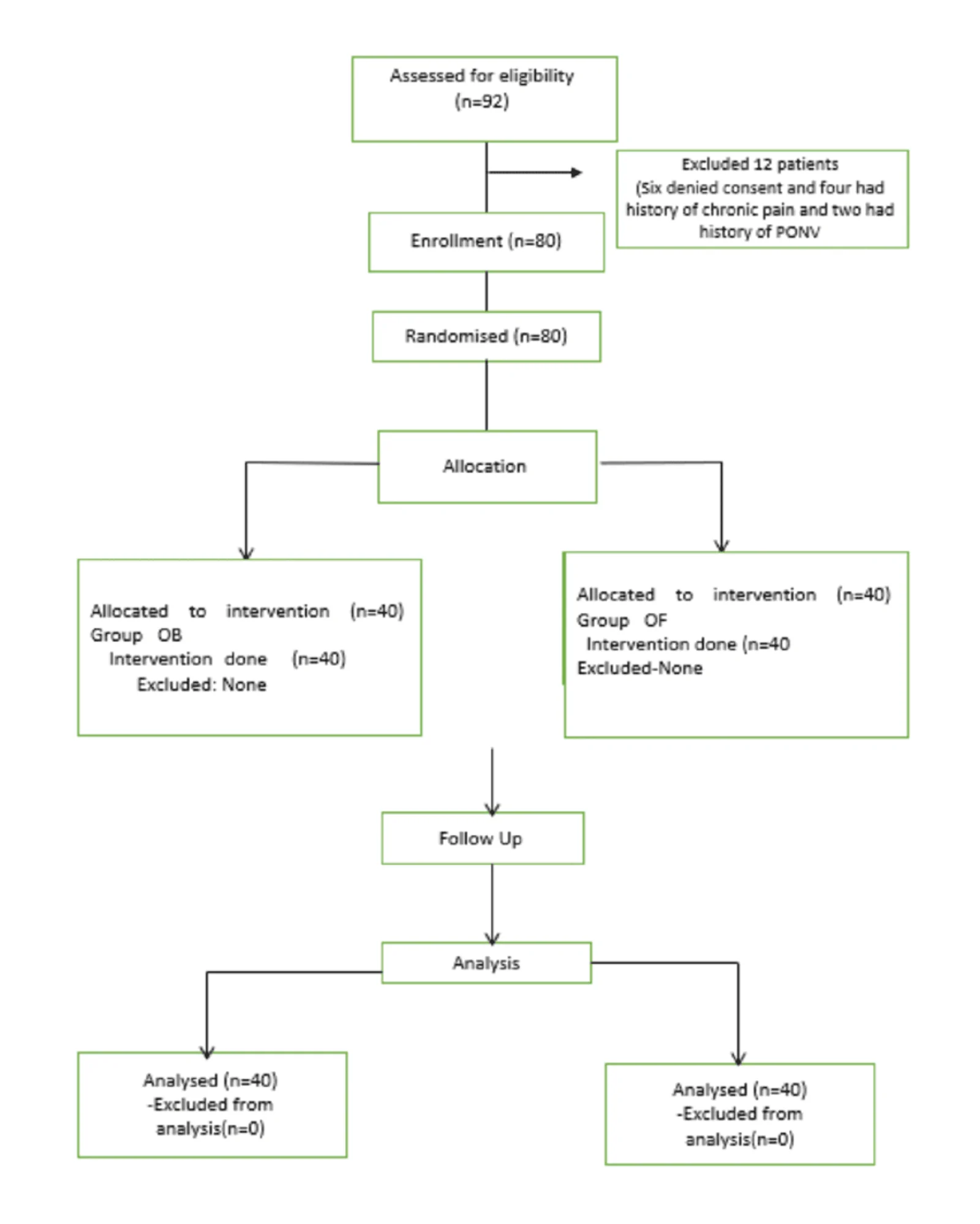
CONSORT diagram CONSORT: Consolidated Standards of Reporting Trials

Figure 1 shows the CONSORT (Consolidated Standards of Reporting Trials) flow diagram of the study. Both groups were comparable with respect to age, ASA PS, duration of surgery, preoperative NRS-Pain scores and QoR-40 scores, and baseline hemodynamic parameters (Table 1).

**Table 1:** Comparison of baseline variables between the two groups ^*^OB minus OF OF: opioid-free, OB: opioid-based; CI: confidence interval; SD: standard deviation; ASA PS: American Society of Anesthesiologists-Physical Status; NRS: Numerical Rating Scale; QoR-40: Quality of Recovery-40; SBP: systolic blood pressure; DBP: diastolic blood pressure; MAP: mean arterial pressure; HR: heart rate

Variable	Group-OB (n = 40)	Group-OF (n = 40)	Mean difference^*^,odds ratio (95% CI)	P-value
Age, years, mean (SD)	43.68 (10.67)	44.45 (9.91)	-0775 (-5.36 to 3.81)	0.737
ASA PS I, n (%)	11 (27.5)	9 (22.5)	-	0.606
ASA PS II, n (%)	29 (72.5)	31 (77.5)	-	0.685
Duration of surgery, hours, mean (SD)	206.75 (22.12)	204.75 (21.84)	2.0 (-7.78 to 11.78)	
Pre-NRS-Pain (=1), n (%)	22 (55.0%)	23 (57.5%)	1.11 (0.46 to 2.68)	0.822
SPB baseline, mmHg, mean (SD)	121.0 (13.1)	125.0 (12.8)	-4.0 (-9.71 to 1.76)	0.172
DBP baseline, mmHg, mean (SD)	77.7 (10.5)	80.2 (9.5)	-2.6 (-7.0 to 1.9)	0.259
MAP baseline, mmHg, mean (SD)	93.0 (11.4)	95.4 (9.9)	-2.4 (-7.1 to 2.4)	0.394
HR baseline, bpm, mean (SD)	83.5 (11.3)	84.0 (10.1)	-0.6 (-5.1 to 4.0)	> 0.05

In the present study, the QoR-40 scores were consistently higher in the OF group than in the OB group at all postoperative time points. Linear mixed-effects modeling revealed a significant overall group effect and a significant group × time interaction, with adjusted mean differences ranging from 3.9 to 6.0 points, indicating improvements that approached or met the minimal clinically important difference (Table 2). The minimal clinically important difference (MCID) for the QoR-40 has been reported as 6.3 points [10].

**Table 2:** QoR-40 at different time points ^*^Statistically significant SD: standard deviation; CI: confidence interval; OB: opioid-based; OF: opioid-free; QoR-40: Quality of Recovery-40

Variable	Mean (SD)		Median (25th to 75th percentile)		Adjusted mean difference (95% CI)	P-value (mixed-effect model)
	Group-OB (n = 40)	Group-OF (n = 40)	Group-OB (n = 40)	Group-OF (n = 40)		
QoR-40, 12th hour	152.4 (5.77)	158.4 (5.0)	149.5 (148.0–157.0)	159.5 (155.0–162.0)	6.0 (3.8–8.2)	< 0.001^*^
QOR-40, day 1	159.8 (4.0)	163.7 (5.2)	158.5 (157.0–163.0)	164.5 (157.0–163.0)	3.9 (1.7–6.1)	0.0011^*^
QoR-40, day 3	164.8 (5.80)	170.8	166.5 (163.3–168.0)	170.0 (169.0–173.8)	6.0 (3.5–8.5)	< 0.001^*^

On comparison of postoperative NRS-Pain scores, no significant difference at any time point was observed between the two groups. At the 12th and 24th hour, the mean NRS score was higher in the OB group; however, this difference was not significant (Table 3). None of the patients required intraoperative fentanyl administration.

**Table 3:** Comparison of NRS-Pain scores between the groups at different time points OB: opioid-based; OF: opioid-free; NRS: Numerical Rating Scale

NRS-Pain score	Median (25th percentile to 75th percentile)		P-value	Bonferroni-adjusted p-value
	Group OB (n = 40)	Group OF (n = 40)	P-value	
0 hour	1 (1–2)	2 (1–2)	0.690	1.00
0 to 2nd hour	2 (2–2)	2 (2–2)	0.969	1.00
2nd to 4th hour	2 (2–2)	2 (2–2)	0.796	1.00
4th to 8th hour	3 (3–3)	3 (3–3)	0.723	1.00
8th to 12th hour	3 (3–4)	3 (2–3)	0.217	1.00
12th to 24th hour	3.5 (2–4)	2 (2–4)	0.014	0.084

The rescue analgesic (inj. diclofenac) use in the first 24 hrs was observed to be comparable between the two groups at most of the time points; however, at 16th-20th hour, it was significantly higher in the OB group when compared to the OF group (Table 4).

**Table 4:** Rescue analgesic requirement at different time points ^*^Statistically significant OB: opioid-based; OF: opioid-free

Rescue analgesic consumption	N (%)		P-value	Bonferroni-adjusted p-value
	Group OB (n = 40)	Group OF (n = 40)	P-value	
0 to 4th hour	0 (0.0)	0 (0.0)	-	--
4th to 8th hour	0 (0.0)	0 (0.0)	-	--
8th to 12th hour	3 (7.5)	0 (0.0)	0.241	1.00
12th to 16th hour	16 (41.0)	5 (12.5)	0.004^*^	0.016^*^
16th to 20th hour	16 (41.0)	2 (5.0)	< 0.001^*^	0.001^*^
20th to 24th hour	2 (5.0)	0 (0.0)	0.494	1.00

The incidence of PONV was significantly higher in the initial eight hours in the OB group and was comparable at the rest of the time points between the two groups (Table 5). The time to ambulation, oral fluid tolerance, and hospital stays were observed to be comparable between the two groups (Table 6).

**Table 5:** Comparison of PONV incidence between the two groups ^*^Statistically significant OB: opioid-based; OF: opioid-free; PONV: postoperative nausea and vomiting

PONV	N (%)		P-value	Bonferroni-adjusted p-value
	Group OB (n = 40)	Group OF (n = 40)	P-value, Chi-square (unadjusted)	
0 to 4th hour	33 (82.5)	0 (0.0)	< 0.001^*^	< 0.001^*^
4th to 8th hour	16 (40.0)	0 (0.0)	< 0.001^*^	< 0.001^*^
8th to 12th hour	9 (22.5)	4 (10.0)	0.130	0.780
12th to 16th hour	8 (20.0)	4 (10.0)	0.210	1.00
16th to 20th hour	20 (50.0)	9 (22.5)	0.011^*^	0.066
20th to 24th hour	9 (22.5)	5 (12.5)	0.239	1.00

**Table 6:** Comparison of time to ambulation, time to oral fluid tolerance, and hospital stay between the two groups SD: standard deviation; OB: opioid-based; OF: opioid-free

Parameters	Group OB (n = 40)	Group OF (n = 40)	P-value, Chi-square (unadjusted)
Time to ambulation, seconds, mean (SD)	6050 (648.7)	6097 (682.1)	0.751
OF tolerance, seconds, mean (SD)	5526.6 (1040.8)	5709.9 (820.5)	0.384
Hospital stay, median (25th to 75th percentile)	6.0 (6.0–7.0)	6.0 (6.0–7.0)	0.381

The intraoperative hemodynamics remained comparable between the two groups. In the OF group, 5% (n = 2) of patients experienced hypotension, and only 2.5% (n = 1) of patients in the OB group had bradycardia. These events were successfully managed by adjusting the dexmedetomidine infusion dose, with no additional intervention. However, only 2.5% (n = 1) of patients in the OB group had hypotension, which was successfully managed with IV fluids and a single dose of 3 mg of injectable mephentermine. No other significant side effects related to opioid or non-opioid drugs, such as dexmedetomidine and ketamine, were observed in either group.

## Discussion

A significantly improved QoR was observed in the immediate postoperative period in patients receiving OFA for gynecological cancer surgeries. Similarly, postoperative analgesia was better, and the incidence of early PONV was significantly reduced in the OFA group. In the present study, we used a PROM, in the form of QoR scores, as the primary measure of the therapeutic efficacy of OFA. The QoR-40 questionnaire, developed by Myles et al. [11], is one of the most widely used and validated instruments for assessing patients' early postoperative quality of recovery and overall health status. The QoR evaluation considers the patient’s postoperative status relative to their preoperative status and includes the return of self-care, household and work activities, and mobility to preoperative levels.

Studies evaluating OFA with QoR as the primary outcome have been conducted in breast cancer surgeries [8,9]. Hontoir et al. [8] used the QoR-40, and Zhang et al. [9] used the QoR-15 questionnaires for this indication. Hontoir et al. reported a statistically significant difference in QoR-40 scores, especially in the domains of emotional support and pain, 24 h after surgery [8]. Zhang et al. reported a QoR-15 global score of 140.3 ± 5.2 in the OFA group and 132.0 ± 12.0 in the control group (p < 0.001). The percentage of patients with good recovery (QoR-15 global score ≥ 118) was 100% (40/40) in the OFA group and 82.5% (33/40) in the control group (p = 0.012) [9]. In both of the aforementioned studies, there was no difference in adverse events. Hontoir et al. reported improved sedation at 60 min in the PACU; however, there was no difference in the length of stay.

On comparison of postoperative NRS pain scores, no significant difference was observed at any time point between the two groups. At the 12th and 24th hours, the mean NRS score was higher in the OB group; however, the difference was not significant. Although pain scores were similar between the groups, the greater need for rescue analgesics in the OB group indicates that OFA offered more consistent baseline analgesia and decreased reliance on additional analgesic interventions. Our finding contrasts with that of a previous study, which reported superior postoperative analgesia following breast cancer surgery with the OFA group requiring less opioid consumption [8]. Similar to our study, Rangel et al. [15], in patients undergoing prostate cancer surgery, observed no difference in morphine consumption, despite a greater proportion of patients in the OFA group experiencing moderate to severe pain in the PACU. The results of all these studies suggest that the potential benefits of OFA in terms of analgesia still need to be validated in cancer surgeries. Since pain is a major and integral component of the quality of recovery in perioperative care, studies focusing on postoperative pain and opioid consumption are needed, particularly in specific types of cancer surgeries, since they may inherently be associated with different pain intensities.

In addition to its single-center design, this study has several other limitations that merit consideration. Firstly, the administration of continuous postoperative epidural analgesia would have been desirable rather than intermittent epidural top-ups of isobaric bupivacaine 0.5%, but this was not feasible due to logistic constraints. Secondly, the rescue antiemetic requirement was not recorded. Thirdly, the study was single-blinded due to the nature of the intervention. Fourthly, long-term outcomes with OFA, such as the incidence of chronic persistent post-surgical pain and parameters like the neuropathic pain component and quality of recovery at later time intervals, were not assessed. Lastly, only the overall QoR-40 score was analyzed. A domain-specific analysis of the QoR questionnaire was not performed, which may have provided more detailed insights into the different dimensions of postoperative recovery.

## Conclusions

The present study demonstrated significantly better postoperative quality of recovery with OFA compared to OBA, with QoR-40 scores being 3.9-6.0 points higher, approaching or achieving the minimal clinically important difference of 6.3 points, in patients undergoing gynecological cancer surgery. In addition, postoperative analgesia and PONV were significantly improved in the OFA group. However, intraoperative and postoperative hemodynamics, time to first independent ambulation, oral fluid tolerance, and hospital stay were comparable between the two groups. Future studies with larger sample sizes and longer follow-up periods are required to validate these findings.
